# Prediction and positioning of UWSN mobile nodes based on tidal motion model

**DOI:** 10.1038/s41598-024-65201-2

**Published:** 2024-07-02

**Authors:** Xiuwu Yu, Dengfeng Li, Yinhao Liu, Ke Zhang, Yong Liu

**Affiliations:** 1 School of Resource Environment and Safety Engineering, University of South China, Hengyang, 421001 China; 2 School of Electrical Engineering, University of South China, Hengyang, 421001 China; 3 College of Physics and Optoelectronic Engineering, Shenzhen University, Shenzhen, 518000 China

**Keywords:** Tidal movement model, TDOA positioning, Niche technology, Genetic algorithm, Kalman filtering, Engineering, Physics, Information theory and computation

## Abstract

As the node positioning of underwater wireless sensor networks is easily affected by tidal motion, ocean current motion and multipath effect, the node positioning accuracy is low. In order to better improve the positioning accuracy of moving nodes of underwater wireless sensor networks, a method of locating mobile nodes of underwater wireless sensor based on tidal motion model is proposed. Firstly, the Time Difference of Arrival (TDOA) localization optimized by niche genetic algorithm is used to initialize each node. The integration of niche technology can effectively find multiple excellent solutions in the solution space, thus providing more abundant solution choices. This algorithm has excellent performance in multi-modal optimization problems, and can avoid the algorithm falling into local optimal solutions, so as to obtain more comprehensive optimization results. The simulation results show that the proposed algorithm has better positioning accuracy than the traditional Chan algorithm and Taylor algorithm. Then, each node is updated in real time by the optimized tidal movement model formula predicted by Kalman filter algorithm. The prediction algorithm is used to compare the real-time predicted update position of the node with the actual position. The positioning distance error of the prediction algorithm is also enough to meet the practical application requirements.

## Introduction

The importance of marine monitoring is closely linked to the application of Underwater Wireless Sensor Networks (UWSN), as together they form the basis for effective management and protection of the marine environment. The ocean not only provides rich biological resources for human beings, but also plays a key role in the stability of global climate^1^. However, with the increasing impact of human activities on the marine environment, such as overfishing. In order to understand marine pollution and climate change, the continuous monitoring of the ocean has become particularly important. As an emerging technology, UWSN provides an effective solution for marine monitoring. The UWSN consists of a large number of distributed sensor nodes that are able to work together to collect various data about the marine environment, such as temperature, salinity, flow velocity, biodiversity, etc. These data are of great value for scientists to understand marine ecosystems, predict climate change, manage marine resources, and respond to marine disasters^2^. However, the implementation of UWSN faces many challenges, one of which is the localization problem of underwater nodes. Due to the particularity of the underwater environment, such as attenuation of signal propagation, multipath effect, and the complexity of the underwater environment, accurate positioning becomes difficult^3^. In addition, the deployment and maintenance of underwater devices are costly, which also increases the complexity of the localization problem. To address these issues, researchers are developing new localization algorithms and techniques, such as sound-based localization methods, inertial navigation systems, and localization techniques that exploit the behavior of marine organisms. Accurate node localization can not only improve the accuracy of data collection, but also optimize the energy consumption of the network and prolong the life cycle of the network. This is essential for long-term monitoring in the vast and harsh marine environment. By improving the positioning accuracy, UWSN can more effectively serve many fields such as marine scientific research, resource exploitation, environmental protection and disaster prevention, thus contributing to the sustainable development of human society. Therefore, continuous research and technological innovation is the key to promote the development and improvement of UWSN, and it is also the basis for accurate marine monitoring.

## Related works

Zhang et al.^4^ proposed a node Mobility Prediction Localization (MPL) algorithm for UWSN. They used a hierarchical optimization strategy and node position prediction to design the localization algorithm, which first selects a buoy node as the initial node and periodically broadcasts ranging requests. The node receives the request and determines the node location through TOA. The node then predicts the speed and position of the node at this time according to the speed at the previous time. At the same time, the GWO algorithm is used to optimize the secondary nodes, so as to optimize the node position and improve the positioning accuracy of the node. However, if the node is affected by unexpected water flow or other factors, the accuracy of prediction may decrease. Chizari^5^, Saeed^6^ and Liu^7^ et al. proposed a Approximate Convex Decomposition Localization (ACDL) algorithm, which uses beacons to assist localization and can move vertically. The location is obtained by communicating with the GPS of the surface buyable, and then the location of different depths is broadcast to reduce the communication distance, so as to expand the positioning coverage. However, if the nodes in the network move or a new node join, the original convex decomposition may not be effective anymore, and the algorithm needs to be executed again, which may increase additional computational overhead. Wu et al.^8^ proposed a Scalable Localization with Mobility Prediction (SLMP) algorithm. The whole process of the algorithm is divided into anchor node localization and ordinary node localization. When the anchor node is located, it directly communicates with the water surface to realize self-localization. In the localization of ordinary nodes, the movement pattern of this node is inferred from the neighboring nodes and its position is predicted through the spatial correlation of underwater movement characteristics. This algorithm maintains a high localization coverage rate, but the maximum likelihood estimation method has a certain error in calculating the node coordinates, resulting in low localization accuracy. Lv et al.^9^ proposed that the positioning result of Chan algorithm in Ultra Wide Band (UWB) was used as the initial value of Taylor algorithm, which ensured the convergence and computational efficiency of Taylor algorithm. The positioning compensation error was obtained through the preset body length of monorail crane and double tag positioning data, and the error was replaced into Taylor algorithm to further improve the positioning accuracy. However, the weighted C-T fusion algorithm and Unscented Kalman Filter (UKF) optimization processes mentioned in this paper are relatively complex and may require high computational resources and processing power, which may lead to real-time problems in practical applications. Zhang et al.^10^ proposed a multi-step localization strategy based on Mobility Prediction and Particle Swarm Optimization (MP-PSO). Firstly, the range-based PSO algorithm is used to calculate the anchor node's immediate velocity by obtaining the localization results of the anchor node at two times, and then the velocity of the unknown node is estimated by combining the spatial correlation of the underwater object. The energy consumption of the algorithm is considered to significantly reduce the average communication cost of the network through the mobility prediction scheme. However, the positioning of ordinary nodes depends on the accurate positioning and prediction of anchor nodes, and the robustness of the algorithm is insufficient. Yan et al.^11^ proposed the application of Chan algorithm in the field of maritime sound source localization, which uses an efficient non-iterative localization method and solves the Time Difference Of Arrival (TDOA) equations by the least square method, which is suitable for maritime sound source localization and has high positioning accuracy and computational efficiency. However, the ill-conditioned matrix problem may be encountered under specific conditions, which affects the accuracy and stability of positioning results. Jiang et al.^12^ proposed a joint localization method based on Taylor weighted least squares algorithm. The position and velocity of the target were initially estimated by the weighted least squares method, and then the Taylor expansion was used for iterative optimization until it approached the Cramer-Rao bound, which improved the localization accuracy in low noise conditions, but the performance may decline in high noise environments. Li et al.^13^ proposed a new short-wave TDOA positioning algorithm based on joint optimization of genetic algorithm. By jointly optimizing ionospheric reflection virtual height and target position through genetic algorithm, accurate positioning can be achieved without ionospheric parameter information, but more iterations are needed to ensure accuracy when ionospheric conditions change greatly. Based on the above research, in order to further accurately locate the underwater mobile nodes and reduce the communication overhead, this paper proposes an underwater environment based on the tidal movement model^14^ and uses the niche genetic algorithm^15^ to improve the TDOA positioning algorithm to initialize the position of the node. Then Kalman Filter (KF) algorithm^16^ is used to predict and update the model parameters, and the position of each node is predicted, which effectively improves the positioning accuracy, reduces the energy consumption of the node and prolongs the lifetime of the node.

## Tidal movement model

**Figure 1:**
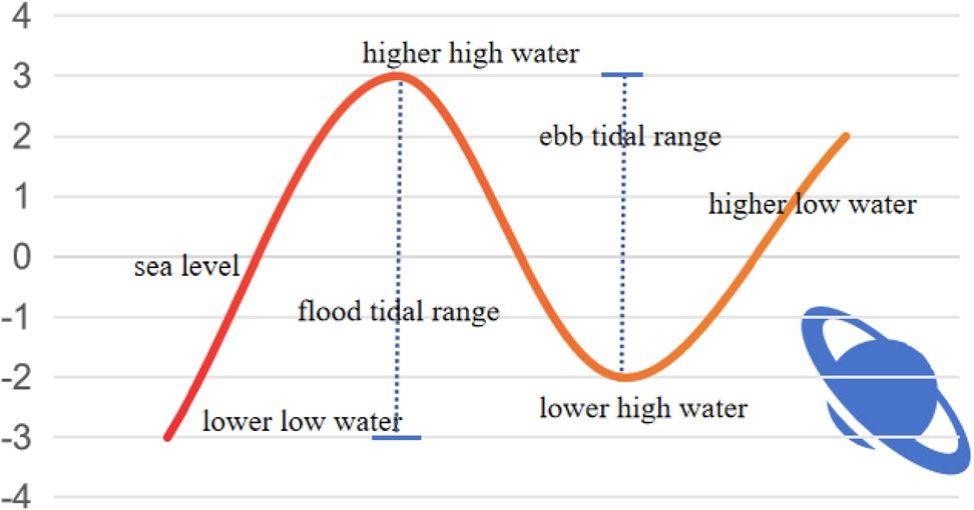
Tidal movement model.

Tidal motion models are mathematical models used to describe tidal phenomena on the earth. Tides are periodic fluctuations in the ocean caused by gravitational interactions. The goal of tidal motion models is to predict and explain the occurrence and change of tides. In the offshore area, the main factor affecting the location accuracy of nodes is tidal movement. The physical model of tidal movement is shown in Fig. 1:

The sea rises during the day and at night, with high tides and low tides.

The mathematical expression of the tidal movement model is as follows:

1 $$v(x,t) = v_{0} (x) + \sum\limits_{i = 1}^{N} {\left[ {p_{i} (x)\cos (w_{i} t)} \right]} + \sum\limits_{i = 1}^{N} {\left[ {q_{i} (x)\sin (w_{i} t)} \right]}$$

In the formula, *x* represents the position parameter, *v* represents the velocity at time *t* at the *x* position, *N* represents the number of tidal components, cos(*w*_*i*_*t*) and sin(*w*_*i*_*t*) represent the time base function of the tidal components at frequency *w*_*i*_, and *v*_*0*_(*x*) represents the average velocity of the ocean current during the sampling interval. *p*_*i*_(*x*) and *q*_*i*_(*x*) are both position-dependent functions.

Considering that the node motion caused by tidal motion is nonlinear, and the motion perpendicular to the direction of water flow is a finite damping motion, and considering that the Gaussian radial basis function has small error and high smoothness, the Gaussian radial basis function is adopted as the spatial basis function^17^. In Eq. (1), *v*_0_ (*x*), *p*_*i*_(*x*) and *q*_*i*_(*x*) can be expressed as:

2 $$v_{0} (x) = \sum\limits_{j = 1}^{M} {\mu_{1,j} } \psi {}_{j}(x)$$

3 $$p_{i} (x) = \sum\limits_{j = 1}^{M} {\mu_{2i,j} \psi_{j} (x)}$$

4 $$q_{i} (x) = \sum\limits_{j = 1}^{M} {\mu_{2i + 1,j} \psi_{j} (x)}$$

5 $$\psi_{j} (x) = \exp (\frac{{ - ||x - c_{j} ||^{2} }}{{2\sigma^{2} }})$$

In the above equation, *μ*_*i,j*_ is the coefficient of the Gaussian radial basis function, *M* represents the number of Gaussian radial basis functions, *c*_*j*_ is the center position of the jth Gaussian radial basis function, *σ* represents the width of the Gaussian radial basis function. Substituting Eqs. (2), (3) and (4) into Eq. (1) yields:

6 $$\begin{gathered} v(x,t) = \sum\limits_{j = 1}^{M} {\mu_{1,j} } \psi {}_{j}(x) + \sum\limits_{i = 1}^{N} {\left[ {\sum\limits_{j = 1}^{M} {\mu_{2i,j} \psi_{j} (x)} \cos (w_{i} t)} \right]} \hfill \\ + \sum\limits_{i = 1}^{N} {\left[ {\sum\limits_{j = 1}^{M} {\mu_{2i + 1,j} \psi_{j} (x)} \sin (w_{i} t)} \right]} \hfill \\ \end{gathered}$$

7 $${\text{If}}\quad \gamma_{j} (t) = \mu_{1,j} + \sum\limits_{i = 1}^{N} {\mu_{2i,j} } \cos (w_{i} t) + \sum\limits_{i = 1}^{N} {\mu_{2i + 1,j} } \sin (w_{i} t)$$

8 $${\text{Then}}\quad v(x,t) = \sum\limits_{j = 1}^{M} {\gamma_{j} (t)} \psi {}_{j}(x)$$

It can be seen from the above formula that by inputting the position information of the node at time *t*, the velocity of the node at time *t* is output. The result calculated by this formula is closely related to the Gaussian radial basis function coefficient (*μ*_*i,j*_) and the center position of the jth Gaussian radial basis function (*c*_*j*_). For the anchor node, *M* points are randomly selected as the center of the Gaussian radial basis function in the communication region of the anchor node. For ordinary nodes, the position of the received anchor node is taken as the center position of the Gaussian radial basis function, and then Kalman filter algorithm is used to predict and optimize the Gaussian radial basis function coefficient (*μ*_*i,j*_). Thus, the node motion equation can be updated in real time.

## Kalman filter algorithm prediction and optimization

In order to use the Kalman filtering algorithm to predict and optimize the coefficient of the motion model $\mu_{i,j}$, assuming that the positioning period of the node is $P$, $t_{k} = k \times P$ then Eq. (7) can be rewritten as:

9 $$\gamma_{j} (kP) = \mu_{1,j} + \sum\limits_{i = 1}^{N} {\mu_{2i,j} } \cos (w_{i} kP) + \sum\limits_{i = 1}^{N} {\mu_{2i + 1,j} } \sin (w_{i} kP)$$

Taylor expansion of $\cos (w_{i} kP)$ and $\sin (w_{i} kP)$ yields the following equation:

10 $$\begin{gathered} \gamma_{j} (kP) = \gamma_{j} \left[ {(k - 1)P} \right] + \hfill \\ \sum\limits_{i = 1}^{N} {w_{i} P\left[ {\mu_{2i,j} ,\mu_{2i + 1,j} } \right]\left[ \begin{gathered} - \sin (w_{i} (k - 1)P) \hfill \\ \cos (w_{i} (k - 1)P) \hfill \\ \end{gathered} \right]} \hfill \\ \end{gathered}$$

If $\gamma_{k}$ is used to represent $\gamma (kP) = \left[ {\gamma_{1} (kP),\gamma_{2} (kP), \cdots ,\gamma_{M} (kP)} \right]^{T}$, the state equation of Kalman filtering algorithm can be expressed as:

11 $$\gamma_{k} = \gamma_{k - 1} + \sum\limits_{i = 1}^{N} {w_{i} P \left[ \begin{matrix} {{\mu _{2i,1}}} & {{\mu _{2i + 1,1}}} \\ \vdots & \vdots \\ {{\mu _{2i,M}}} & {{\mu _{2i + 1,M}}} \\ \end{matrix} \right] \left[ \begin{gathered} - \sin (w_{i} (k - 1)P) \hfill \\ \cos (w_{i} (k - 1)P) \hfill \\ \end{gathered} \right]} + w(k)$$

where $w(k) = [\omega_{1} (k),\omega_{2} (k), \ldots ,\omega_{M} (k)]^{T}$ represents the noise error at time *t*, assuming $w(k)$ is Gaussian white noise, its mean is 0, and the covariance matrix is known.

The observation equation of Kalman filter algorithm is as follows:

12 $$z_{k} = H\gamma_{k} + \delta (k)$$

where $H = [\psi_{1} (x),\psi_{2} (x), \cdots ,\psi_{M} (x)]$, $\delta (k)$ represents the observed noise with Gaussian distribution, whose mean value is 0 and whose covariance matrix is known.

According to the observation equation and state equation of Kalman filter algorithm, the optimal state estimation of matrix *γ* can be obtained, and the specific calculation process is as follows:

According to Eq. (11), the state of *γ* at the next time can be predicted:

13 $$\begin{gathered} \gamma_{k|k - 1} = \gamma_{k - 1|k - 1} \hfill \\ + \sum\limits_{i = 1}^{N} {w_{i} P \left[ \begin{matrix} {{\mu _{2i,1}}} & {{\mu _{2i + 1,1}}} \\ \vdots & \vdots \\ {{\mu _{2i,M}}} & {{\mu _{2i + 1,M}}} \\ \end{matrix} \right] \left[ \begin{gathered} - \sin (w_{i} (k - 1)P) \hfill \\ \cos (w_{i} (k - 1)P) \hfill \\ \end{gathered} \right]} + w(k) \hfill \\ \end{gathered}$$

The $\gamma_{{\left. {k - 1} \right|k - 1}}$ in the above formula represents the optimal value of $\gamma$ at time $(k - 1)P$.

The error estimation of the covariance matrix can be expressed as:

14 $$p(k|k - 1) = p(k - 1|k - 1) + Q(k)$$

$Q(k)$ in the above equation represents the covariance matrix of Gaussian white noise $w(k)$.

Kalman gain can be expressed as:

15 $$Kg_{k} = p(k|k - 1)H^{T} /(H \, p(k|k - 1)H^{T} + R(k))$$

The $R(k)$ in the above formula represents the covariance matrix of the observed noise $\delta (k)$ following the Gaussian distribution.

The covariance matrix is then updated by the following formula:

16 $$p(k|k) = (I - Kg_{k} H) \times p(k|k - 1)$$

Finally, the optimal value of $\gamma$ at time $kP$ is:

17 $$\gamma_{k|k} = \gamma_{k|k - 1} + Kg_{k} \times (z_{k} - H\gamma_{k|k - 1} )$$

Thus, the optimal value of the next $\gamma$ at each time can be predicted, and the optimal value of the motion model coefficient $\mu_{i,j} (k)$ can be obtained by the following formula:

18 $$\mu_{i,j} (k) = \left[ {\beta_{k}^{T} \beta_{k} } \right]^{ - 1} \beta_{k}^{T} \gamma_{k|k}^{T}$$

where $\beta_{k} = \left[ {1,\cos (w_{1} kP),\sin (w_{1} kP), \cdots \cos (w_{N} kP),\sin (w_{N} kP)} \right]$, the optimal value prediction of $\gamma$ and $\mu_{i,j}$ at each time can be obtained from the above equation, and the node can update the position of the node in real time by obtaining the speed of the node at a certain time according to the formula (7) and (8).

## Initial node position estimation

**Figure 2:**
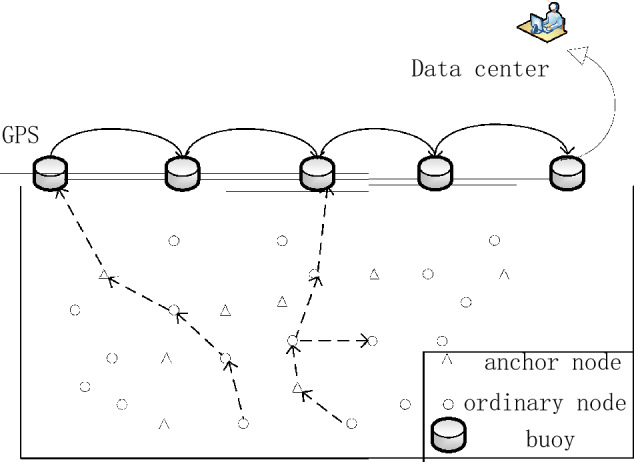
Underwater communication model.

The underwater node communication model is shown in Fig. 2.

### TDOA algorithm

**Figure 3:**
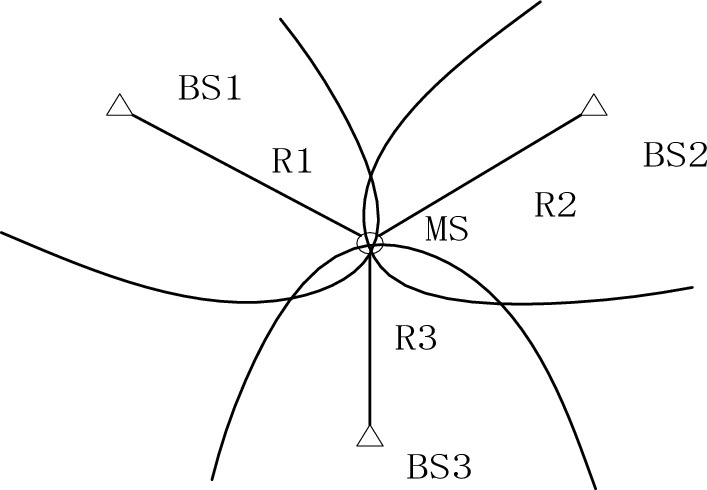
TDOA positioning principle.

TDOA positioning can also be called hyperbolic positioning, its basic principle is a time difference positioning technology, the signal source sends signals, multiple receivers (at least three) receive signals in their respective positions, each receiver receives signals at different time points, record these time points. The relative position of the signal source relative to the different receivers can be calculated by comparing the time difference between the different receivers to receive the signal, and the absolute position of the signal source can be calculated by the relative position of at least three receivers. Its positioning principle diagram is shown in Fig. 3:

First, define the coordinates of $MS$ as $(x,y)$, and the coordinates of $BS_{i}$ as $(x_{i} ,y_{i} )$, $i = 1,2 \cdots ,i$. Then the distance from $MS$ to $BS_{i}$ can be expressed as $R_{i} = \sqrt {(x_{i} - x)^{2} - (y_{i} - y)^{2} }$. If $BS_{1}$ is selected as the service base station, the distance difference between $MS$ to $BS_{i}$ ($i \ne 1$) and to $BS_{1}$ is recorded as $R_{i,1} = R_{i} - R_{1} + vn_{i,1} = vt_{i,1}$, $t_{i,1}$ is the measurement result of TDOA, $v$ is the propagation speed of underwater sound, which is designed in this paper as 1500 m/s, $n_{i,1}$ is the process noise, which is Gaussian white noise subject to normal distribution. Based on the above definition, it can be obtained:

19 $$\begin{gathered} R_{i,1} = \sqrt {(x_{i} - x)^{2} - (y_{i} - y)^{2} } - \\ \sqrt {(x_{1} - x)^{2} - (y_{1} - y)^{2} } + vn_{i,1} \\ \end{gathered}$$

20 $$\overrightarrow {\Delta R} = \left[ {\begin{array}{*{20}c} {R_{21} } \\ {R_{31} } \\ \vdots \\ {R_{i1} } \\ \end{array} } \right] + v\overrightarrow {n}$$

In formula $\overrightarrow {n} = \left[ {n_{2,1} ,n_{3,1} \cdots n_{i,1} } \right]^{T}$, the coordinate position of $MS$ is calculated using maximum likelihood estimation. Since $vt_{i,1}$ is a known quantity and $n$ follows a normal distribution with a mean of 0 and a variance of $\sigma^{2}$, all elements in $\overrightarrow {\Delta R}$ follow a normal distribution with a mean of $R_{i,1}$ and a variance of $\sigma^{2}$. Thus, the corresponding likelihood function can be expressed as:

21 $$L = (\frac{1}{{\sqrt {2\pi } \sigma }})^{i - 1} \prod\limits_{i = 1}^{i - 1} {\exp } ( - \frac{{(\overrightarrow {\Delta R} - \overrightarrow {R} + \overrightarrow {{R_{1} }} )^{T} (\overrightarrow {\Delta R} - \overrightarrow {R} + \overrightarrow {{R_{1} }} )}}{{2\sigma^{2} }})$$

In formula $\overrightarrow {R} = \left[ {R_{2} ,R_{3} \cdots R_{i} } \right]^{T}$,$\overrightarrow {{R_{1} }} = \left[ {R_{1} ,R_{1} , \cdots R_{1} } \right]^{T}_{(i - 1) \times 1}$, the coordinates that make the maximum likelihood estimate the maximum probability are obtained, namely:

22 $$(x,y) = \arg \left\{ {\min } \right.\left. {\left[ {(\overrightarrow {\Delta R} - \overrightarrow {R} + \overrightarrow {{R_{1} }} )^{T} (\overrightarrow {\Delta R} - \overrightarrow {R} + \overrightarrow {{R_{1} }} )} \right]} \right\}$$

The formula contains a very complex nonlinear function, which is difficult to be solved by the traditional derivation method. Since genetic algorithm has no specific requirements on the optimization function, this method is used to find the optimal solution in the whole solution space. In order to prevent the genetic algorithm from falling into a local optimal solution and unable to search for a better solution, the niching technology is introduced to prevent the algorithm from falling into a local optimal situation due to incomplete optimization.

### Niche genetic algorithm (NGA)

#### Genetic algorithm

Genetic Algorithm (GA) is an optimization algorithm inspired by the principles of heredity and evolution in biology. It simulates the process of selection, heredity and variation in biological evolution to achieve the improvement of individual adaptability for problem solving optimization and search. The basic idea is to gradually improve the solution by simulating the evolutionary process. Compared with traditional optimization algorithms, it has the characteristics of non-determinism, self-organization, self-adaptability and self-learning. When chromosomes combine, the combination of genetic factors of both parents allows the child to maintain the characteristics of the parent, and the mutation operation causes the offspring to differ from the parent.

#### Niching technology

Niche technology is an optimization algorithm inspired by the concept of niche in ecology. It simulates the niche distribution and competitive behavior of organisms in nature for solving complex optimization problems. In niche technology, the solution space is viewed as an ecosystem where each solution corresponds to an ecological niche. Each niche has its own specific organizational function, similar to how organisms occupy specific habitats in nature. These niches compete with each other while maintaining a certain distance from other niches to maintain population diversity and prevent local convergence. The goal of niche technology is to discover multiple excellent solutions by maintaining and protecting different ecological niches while searching in different ecological niches. By introducing fitness sharing mechanism, it divides similar solutions into the same niche and competes and evolves within each niche. The basic principle of niche technology is to divide the solutions in a population into different niches based on the similarity or distance between the solutions. Within each subset, the solutions compete and evolve to select the best solution and generate new solutions. Fitness sharing mechanism enables each niche to maintain a certain diversity, and excellent solutions can be better protected and inherited. By maintaining multiple niches, niches technology is able to find multiple good solutions in the solution space, thus providing a richer choice of solutions. It performs well in multi-modal optimization problems and can avoid falling into local optimal solutions, so as to obtain more comprehensive optimization results.

#### Algorithm step

**Figure 4:**
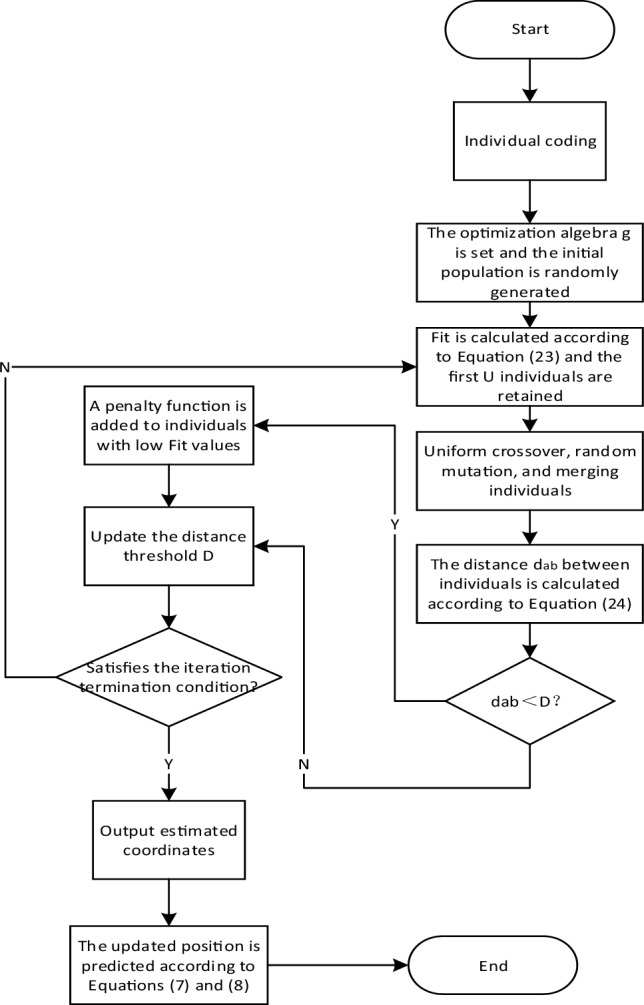
Algorithm flow chart.

Niche technology is introduced into genetic algorithm to form niche genetic algorithm. Its basic idea is as follows: the distance between individuals is first set to D. If the distance between the two individuals is less than D, a penalty function will be assigned to the individual with less fitness to reduce the genetic probability of the individual in the future. In this way, only one excellent individual can be maintained within D, thus avoiding the problem of local optimality caused by excessive closeness of the excellent individuals. The global optimization capability of genetic algorithm is improved, and its basic steps are as follows:

1. Individual coding, binary coding chromosome vector $(x,y)^{T}$, *x, y* represents the possible coordinates of the mobile station.
2. Set the optimized algebra *g*, randomly generate *Q* initial individuals to form the initial population, and calculate the fitness of each individual. The fitness function is as follows:
  23 $$Fit = \frac{1}{{(\overrightarrow {\Delta R} - \overrightarrow {R} + \overrightarrow {{R_{1} }} )^{T} (\overrightarrow {\Delta R} - \overrightarrow {R} + \overrightarrow {{R_{1} }} )}}$$
  The larger the individual value, the higher the fitness of the individual. The value of the individual is arranged in descending order, and the first *U* better individuals are retained.
3. The roulette strategy is used to ensure that the probability of an individual being selected is proportional to the value of $Fit$.
4. Using uniform crossover, randomly generate a binary number that is the same number as the individual code, and select the parent gene according to the value at its corresponding position.
5. The method of random variation is adopted to replace the original gene value of the chromosome with a small probability.
6. Combine the first *U* individuals with high fitness and the individuals obtained after mutation to obtain a new population containing *Q* + *U* individuals, and calculate the distance between each individual in the new population:
  24 $$d_{ab} = \left\| {x_{a} } \right.\left. { - x_{b} } \right\| = \sqrt {\sum\limits_{k = 1}^{i - 1} {(x_{ak} - x_{bk} )^{2} } }$$
  $a = 1,2, \cdots ,Q + U - 1$; $b = a + 1,a + 2, \cdots Q + U$, Where *i-*1 represents the dimension of each individual, $x_{a}$ and $x_{b}$ represent the eigenvectors of individuals $a$ and $b$. When $d_{ab} < D$, a penalty function is assigned to individuals with low fitness in individuals $x_{a}$ and $x_{b}$ to reduce fitness, and *U* individuals with high fitness in the new population are selected to update the niche radius D, whose update rule is the ratio of the average distance between all individuals and algebra.
7. If the preset termination condition is not met, the first *Q* individuals in step (6) are selected to be transferred to step (3), and then the algorithm is iterated until the termination is met, the estimated coordinates are output, and the algorithm is ended. The algorithm flow is shown in Fig. 4:

### Time complexity analysis of the algorithm

For the Niching Genetic Algorithms-Kalman Filter prediction (NGA-KF) algorithm, if the computational complexity of the fitness function is O(f) during the initialization and localization stage of niche genetic optimization TDOA, the time complexity at this stage is O(Q*(*i* − 1)*g + f*Q^2). In the prediction stage using Kalman filter optimization, the time complexity mainly depends on the calculation of the state equation and observation equation, and the algorithm complexity is O(*M*^2). Therefore, the time complexity of the entire algorithm is O(n*(Q*(*i* − 1)*g + f*Q^2 + *M*^2)).

For the MP-PSO algorithm, the PSO algorithm is used for two-dimensional localization during the anchor node localization stage, with a time complexity of O(2*Q*g). In the unknown node localization stage, it depends on the speed and movement prediction of the anchor node, and the time complexity is consistent with the anchor node localization stage. Therefore, the time complexity of the entire algorithm is O(2*n*Q*g).

For the MPL algorithm, the time complexity for locating secondary nodes during the optimization of TOA positioning using GWO algorithm is O(Q*g). The time complexity in the mobile prediction stage is related to the size of the prediction window and the number of nodes, and its time complexity is O(n*kP). Therefore, the time complexity of the entire algorithm is O(n*(Q*g + *kP*)).

For the SLMP algorithm, the future position of anchor nodes is predicted using temporal correlation based on underwater mobility characteristics during the anchor node positioning stage, with a time complexity of O((0.2n)^2*g). In the ordinary node positioning stage, the position is predicted based on the information of its neighboring nodes, with a time complexity of O(0.8n*M). Therefore, the time complexity of the entire algorithm is O((0.2n)^2*g + 0.8n*M).

## Simulated analysis

### Analysis of initial node positioning accuracy

**Figure 5:**
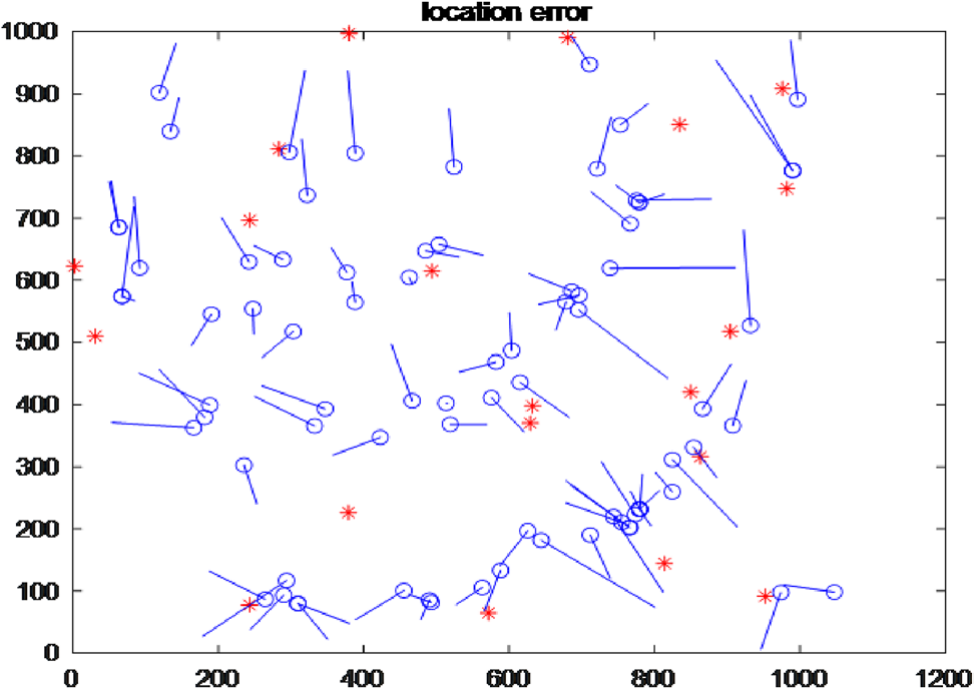
Positioning result.

In this paper, the accuracy of the positioning algorithm is verified by Matlab simulation platform. The depth of the node is obtained by the pressure sensor of the node. The three-dimensional problem is transformed into a two-dimensional problem, and the area of 1000 × 1000 is taken to simulate the tidal environment. When 100 nodes are deployed in the monitoring area, including 80 unknown nodes and 20 anchor nodes, the communication radius of nodes is 200 m, and the underwater acoustic communication speed is 1500 m/s, the population number of the NGA algorithm is set to 100, and The optimization algebra is 60 times, crossover rate $p_{c} = 0.5$, mutation rate $p_{m} = 0.05$, and penalty parameter $penalty = 10^{ - 7}$. TDOA improved by NGA algorithm was used to initialize node positioning, and the positioning results were shown in Fig. 5.

Blue “-” indicates the positioning error of the unknown node (connecting the estimated position and real position of the unknown node). The positioning error of the above figure is 0.20915 according to the simulation platform.

In order to determine whether the NGA algorithm can effectively improve the positioning accuracy of nodes, the simulation experiment mainly observes the influence of the proportion of anchor nodes and the communication radius on the average positioning error, and the average positioning error is calculated using the following formula:

25 $$error = \frac{1}{n}\sum\limits_{i = 1}^{n} {\sqrt {(x_{i} - x_{i}^{\prime} )^{2} + (y_{i} - y_{i}^{\prime} )^{2} } }$$

In the formula, $(x_{i} ,y_{i} )$ represents the real position of the node, $(x_{i}^{\prime} ,y_{i}^{\prime} )$ represents the estimated position of the node, and *n* represents the number of unknown nodes. In order to make the experimental effect have a better comparison effect, the traditional Chan algorithm and Taylor algorithm are selected for comparison test, and then the test results are analyzed.

#### Effect of anchor node ratio on positioning accuracy

**Figure 6:**
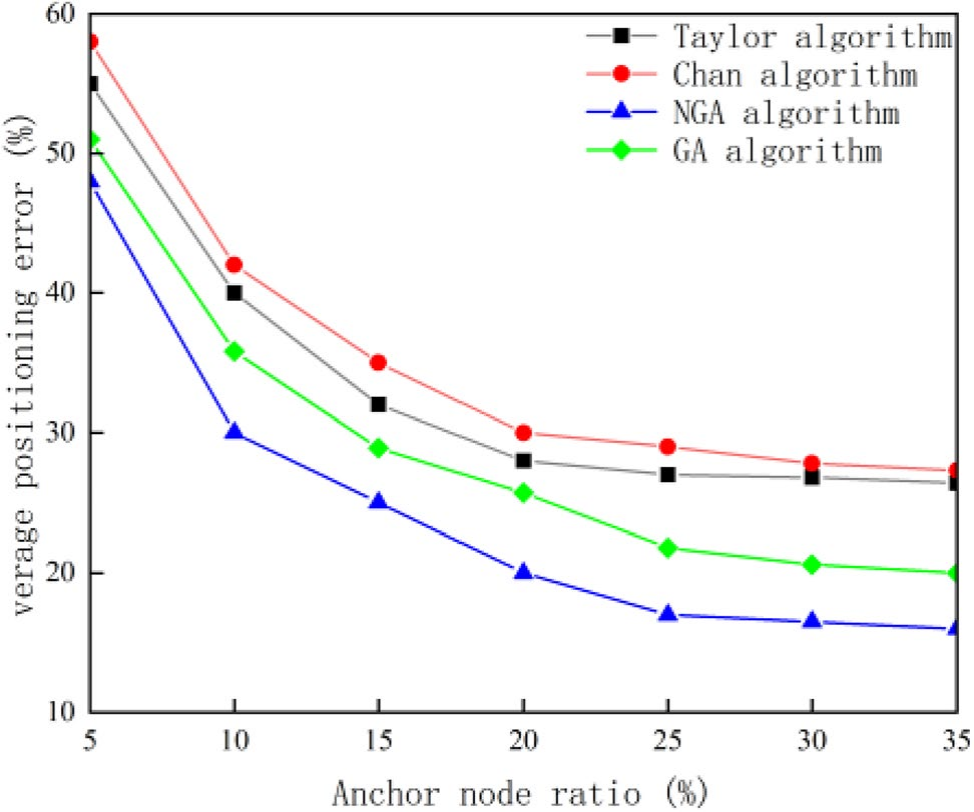
Relationship between the proportion of anchor nodes and the average positioning error.

The 100 wireless sensor nodes are randomly arranged in the area to be monitored, the communication radius of the nodes is set to 300 m unchanged, and the proportion of anchor nodes is 5%, 10%, 15%, 20%, 25%, 30% and 35%, respectively. The simulation results are shown in Fig. 6.

As shown in the figure above, with the increasing proportion of anchor nodes, the average positioning errors of the four algorithms are gradually decreasing. According to the implementation steps of niche genetic algorithm TDOA, increasing the number of anchor nodes can provide more measurement data for the algorithm, increase multiple time difference combinations, and reduce the shadow of multi-path effects in underwater environment, which can reduce positioning errors and improve the robustness of measurement. At the same time, it is not difficult to see from the figure above that when the proportion of anchor nodes is 20%, the average positioning error is as follows in order from large to small: Chan algorithm, Taylor algorithm, NGA algorithm and GA algorithm, which indicates that the positioning accuracy of NGA algorithm is better than the other three traditional algorithms when the number of anchor nodes is certain. Under the same proportion of anchor nodes, the average positioning error of NGA algorithm is reduced by 12.32% and 10.08% compared with the traditional Chan algorithm and Taylor algorithm, reduced by 4.18% compared with GA algorithm.

#### Influence of communication radius on positioning accuracy

**Figure 7:**
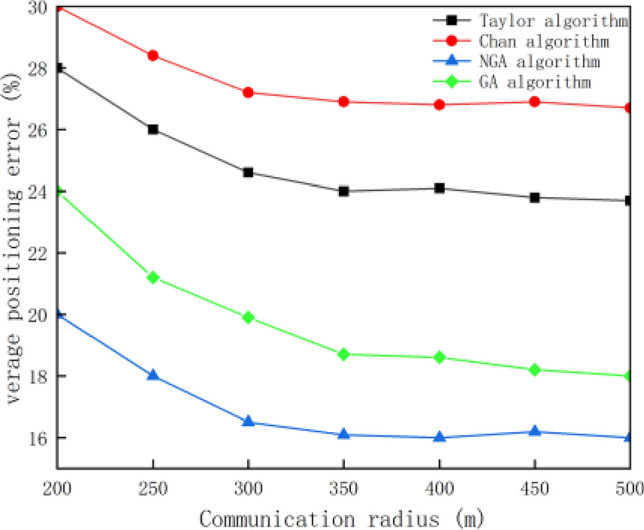
The relationship between communication radius and average positioning error.

Randomly arrange 100 nodes in the area to be monitored, set the proportion of anchor nodes to 20%, and increase the communication radius from 200 to 500 m. The simulation results are shown in Fig. 7.

As can be seen from the figure above, the average positioning error of nodes decreases with the increase of communication radius. When the communication radius is unchanged, the average positioning error of NGA is significantly lower than that of the other three algorithms. When the communication radius is greater than 300 m, the average positioning error of the node tends to change gently, so in order to save node energy and extend the average life of the node, it is not appropriate to consume too much communication energy, and the appropriate communication energy should be used to pursue the improvement of positioning accuracy. Under the same small communication radius condition, the average positioning error of NGA algorithm is reduced by 10.81% and 7.88% compared with the traditional Chan algorithm and Taylor algorithm, reduced by 2.12% compared with GA algorithm.

### Node location trajectory and error analysis under tidal movement

**Figure 8:**
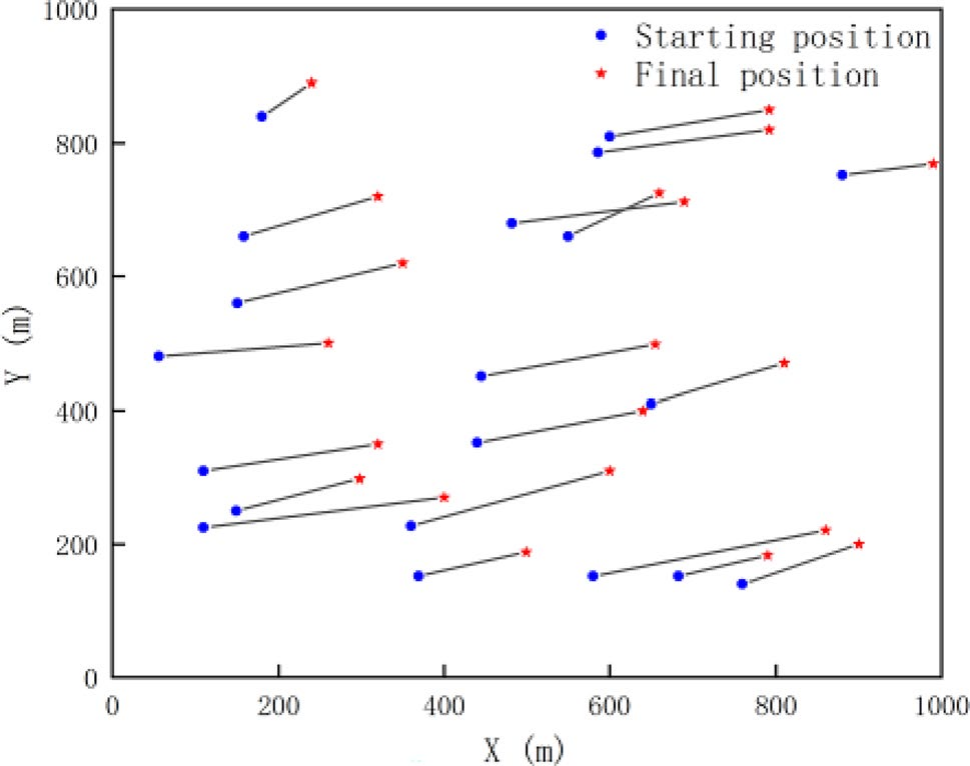
Diagram of node movement under tidal movement.

Considering that in underwater sensor networks, the position movement of nodes is easily affected by tides, ocean currents and other environments, it is likely to lead to the decline of node positioning accuracy. Therefore, in this paper, after the initial positioning of nodes is completed, the velocity of nodes at this moment can be calculated by formula $v(x,t) = \sum\nolimits_{j = 1}^{M} {\gamma_{j} (t)} \psi {}_{j}(x)$ based on the current position information $x$, and the node position can be updated by rule $x^{t + 1} = x + vP$. If the positioning period *P* is selected as 2 s, the node movement during tidal movement simulation is shown in Fig. 8. The figure shows the position changes of 20 nodes after 60 s, in which the blue dot represents the initial position of the node, and the red star represents the new position of the node after 60 s. Through this simulation, the movement of each node under the action of tide can be roughly observed, and the movement of the node can be taken into account in positioning, which can effectively improve the robustness and positioning accuracy of the positioning algorithm.

**Figure 9:**
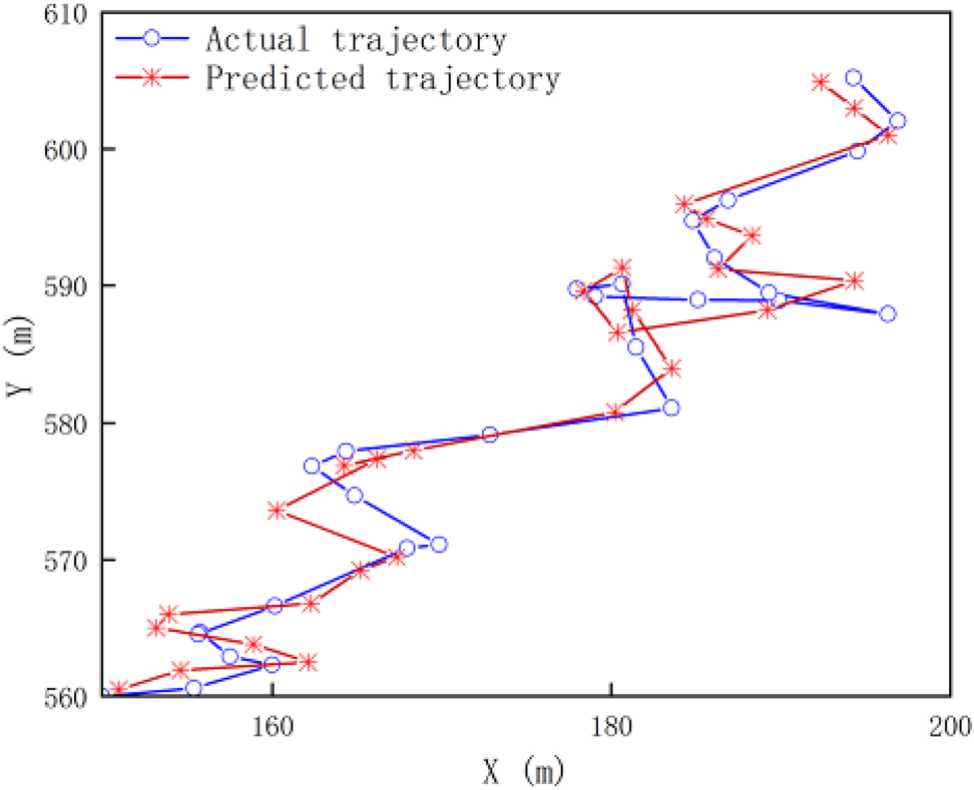
Comparison of real time predicted trajectories with actual trajectories.

**Figure 10:**
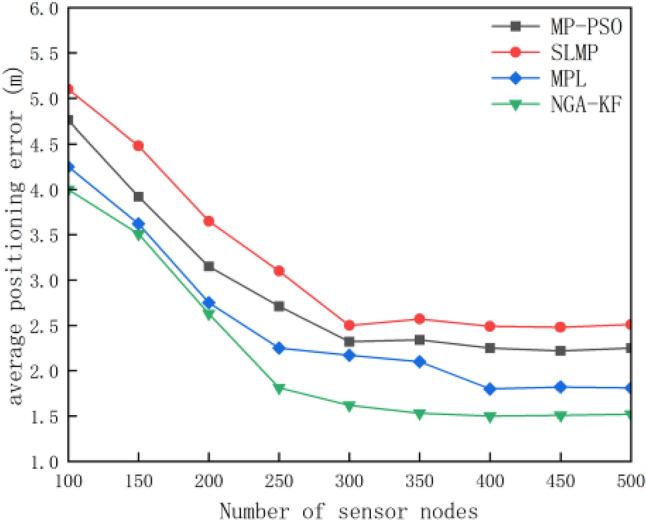
Relationship between node density and average localization error.

The motion information of the anchor node directly affects the positioning accuracy of ordinary nodes. In this paper, an anchor node is randomly selected from the deployed anchor nodes, and the position of the anchor node is predicted and updated in real time within 60 s according to the tidal motion model formula optimized by the Kalman filter algorithm. Figure 9 shows the comparison between the actual trajectory and the predicted trajectory of the anchor node:

In order to understand the positioning advantages of this prediction algorithm more accurately and intuitively, Fig. 10 shows the difference of average positioning errors calculated by different algorithms according to Eq. (25) at a certain time when the proportion of anchor nodes is unchanged:

It can be seen from the above figure that when the node density continues to increase, the average positioning error has a decreasing trend. Compared with MPL^4^, MP-PSO^10^ and SLMP^8^ algorithms, the average positioning error of NGA-KF algorithm is smaller. Compared with the above three algorithms, the positioning error is reduced by 16%, 32%, 39% respectively. With the increase of node density, the number of nodes that provide known information for ordinary nodes increases, including the number of Gaussian radial basis functions (M), which improves the accuracy of node positioning.

## Conclusion

On the basis of traditional TDOA positioning algorithm, combining genetic algorithm and niche technology, a niche genetic algorithm optimized TDOA positioning is proposed. Through simulation experiments, the positioning accuracy of this positioning algorithm has obvious advantages compared with GA algorithm, Chan algorithm and Taylor algorithm. Considering that nodes in underwater environment are vulnerable to the influence of tidal movement and thus reduce the positioning accuracy, Kalman filter algorithm is introduced to predict and optimize the tidal motion model formula to predict and estimate the next moment position of nodes in real time, thereby increasing the robustness and positioning accuracy of positioning. The prediction distance error of this algorithm is sufficient to meet the needs of engineering applications.

## Data Availability

The data that support the findings of this study are available from the corresponding author, Dengfeng Li, upon reasonable request.
